# OX40-OX40L Interaction Promotes Proliferation and Activation of Lymphocytes via NFATc1 in ApoE-Deficient Mice

**DOI:** 10.1371/journal.pone.0060854

**Published:** 2013-04-09

**Authors:** Jinchuan Yan, Hongling Su, Liangjie Xu, Cuiping Wang

**Affiliations:** Department of Cardiology, The Affiliated Hospital of Jiangsu University, Zhenjiang, Jiangsu Province, China; University of Cincinnati, United States of America

## Abstract

**Background:**

Our previous studies have shown that OX40-OX40L interaction regulates the expression of nuclear factor of activated T cells c1(NFATc1) in ApoE^−/−^ mice during atherogenesis. The aim of this study was to investigate whether OX40-OX40L interaction promotes Th cell activation via NFATc1 in ApoE^−/−^ mice.

**Methods and Results:**

The lymphocytes isolated from spleen of ApoE**^−/−^** mice were cultured with anti-CD3 mAb in the presence or absence of anti-OX40 or anti-OX40L antibodies. The expression of NFATc1 mRNA and protein in isolated lymphocytes were measured by real time PCR (RT-PCR) and flow cytometry (FCM), respectively. The proliferation of lymphocytes was analyzed by MTT method,and the expression of IL-2, IL-4 and IFN-γ in the cultured cells and supernatant were measured by RT-PCR and enzyme-linked immunosorbent assary (ELISA), respectively. After stimulating OX40-OX40L signal pathway, the expression of NFATc1 and the proliferation of leukocytes were significantly increased. Anti-OX40L suppressed the expression of NFATc1 in lymphocytes of ApoE−/− mice. Anti-OX40L or the NFATc1 inhibitor (CsA) markedly suppressed the cell proliferation induced by anti-OX40. Moreover, the expression of IL-2 and IFN-γ was increased in lymphocytes induced by OX40-OX40L interaction. Blocking OX40-OX40L interaction or NFATc1 down-regulated the expression of IL-2 and IFN-γ, but didn’t alter the expression of IL-4 in supernatants.

**Conclusion:**

These results suggest that OX40-OX40L interaction promotes the proliferation and activation of lymphocytes through NFATc1.

## Introduction

Atherosclerosis is a chronic-inflammatory disease in the context of hypercholesterolemia, in which both innate and adaptive immune responses play a role [1], [2]. TNF receptor pathway can provide co-stimulatory signals and has been implicated in the onset and progression of atherosclerosis and ACS (acute coronary syndromes). Previous studies from our laboratory and others have shown an emerging role of OX40-OX40L interaction in the development of atherosclerotic lesions during atherogenesis [3]. OX40-OX40L represents a pair of co-stimulatory molecules critical for T cell proliferation, survival, cytokine production, and memory cell generation.

T lymphocytes are present at all stages of atherosclerosis. After signals delivered by antigen (Ag) stimulation and costimulatory signals provided by antigen presenting cells (APCs) during T cell activation, inositol 1.4.5-trisphosphate (IP_3_) induces a rapid increase in intracellular free Ca^2+^. The IP_3_-Ca^2+^ directly binds to nuclear factor of activated T (NFAT) transcription factors in the cytoplasm, resulting in their dephosphorylation and subsequent translocation into the nucleus. This translocation leads to diverse cellular physiological functions, such as secretion, cell proliferation, cell growth, differentiation and aging [4]. NFATc1 is dephosphorylated by a Ca^2+^-dependent serine/threonine phosphatase, calcineurin, and then translocates into the nucleus where they associate with target DNA sequences. The immunosuppressive drugs FK506 and cyclosporine A suppress the function of these NFATs to the same degree through the inhibition of calcineurin activity [5]–[7].

Previous studies showed that blocking the nuclear factor of activated T-cells activation could suppress balloon injury-induced neointima formation [8], [9]. Our recent data show that OX40-OX40L interaction induced a robust stimulation of phospholipase C signal transduction pathway in human endothelial cells [10]. Strikingly, preventing OX40–OX40L interactions inhibited the level of IP3 and intracellular Ca^2+^ mobilization, and the activation of IP3-Ca^2+^ signal pathway was mediated by the interaction of OX40-OX40L. We also find that OX40-OX40L interaction regulated the expression of NFATc1 in ApoE^−/−^ mice [11]. Based on our previous findings and others’ reports, we proposed that NFATc1 was a downstream mediator of OX40-OX40L interaction during atherogenesis.

A whole range of identified cytokines have been shown to play a role in atherogenesis, some with pro-atherogenic properties while others having anti-atherogenic properties. Th cells present in the atherosclerotic lesions showed properties of Th1 phenotype with increased levels of IL-2 and IFN-γ, whereas the Th2 cytokines IL-4 was found in modest quantities [12]. NFAT transcription factors have various implications on many cytokines, such as IL-2, IL-4 and IFN-γ, which are highly associated with NFAT. However, the precise mechanism by which OX40-OX40L interaction target NFATc1 in ApoE^−/−^ mice during atherogenesis remains unclear. Hence, we investigated whether and how OX40-OX40L interaction regulated Th1 cell proliferation and viability through NFATc1 in ApoE^−/−^ mice.

## Methods

### Mice

Male apoE^−/−^ mice on a C57BL/6J background, obtained from the Jackson Laboratory, USA, were housed on a 12 h light–dark cycle in a specific pathogen-free environment with free access to rodent chow and water. All procedures were conducted according to the National Ethics Committee for Care and Use of Laboratory Animals for Research of the Medical School. At 6 weeks of age, the mice were divided into the following groups: control group (sham-operation group) (n = 10), model group (n = 10), OX40/OX40L activated group (n = 10) and OX40/OX40L inhibited group (n = 10). The latter three groups were fed a Western type diet containing 0.25% cholesterol and 15% cocoa butter and a constrictive collar was placed around right carotid arteries of these mice for 4 weeks to induce plaques formation. Then all the mice were euthanized and analyzed.

### Atherosclerotic Plaques Measured by HE Staining

Atherosclerotic plaque sections from the carotid artery (5 µm) were stained with hematoxylin and eosin.

### Cell Culture

24-well plates were coated overnight with anti-CD3 antibody (10 µg/ml; PharMingen, San Diego, CA) at 4°C. Unbound Ab was removed by washing before the addition of cells. 6 weeks old male ApoE−/− mice were killed, and their spleen were removed. Cell suspensions were made by crushing the tissues between gauze. After separation with Ficoll-paque (Amersham Pharmacia Biotech, Herts, UK), the cell suspensions were cultured in 24-well plates at an initial density of 1×10^6^ cells per well. For CD3/OX40 costimulation, cells were cultured with agonistic anti-OX40 mAb(20 µg/ml) in the presence or absence of cyclosporine A (CsA) (A pharmacological inhibitor of the calcineurin-NFAT activation pathway). For soluble blockade using anti-OX40L mAb, cells were cultured with anti-OX40L mAb or inactivated anti-OX40L mAb (20 µg/ml) with anti-OX40 mAb(20 µg/ml). For MTT, cell cultures were harvested without re-stimulation after 24, 48 h and 72 h. For flow cytometric ananlyses, ELISA and real-time PCR, cell cultures were harvested after 72 h.

### The Cell Proliferation Detected by MTT

Lymphocytes were incubate without re-stimulation after 24, 48 h and 72 h, MTT was added at a final concentration of 5 mg/ml, and incubation was continued for a further 4 h. After 4 h, DMSO was added, and following mixing, colorimetric determination of MTT reduction was made at 490 nm.

### Real-Time PCR

Total RNA was isolated from spleen using TRIzol reagent (Invitrogen) according to the manufacturer’s instructions. The RNA was quantified, and reverse-transcribed (RevertAid M-MuLV reverse transcriptase) according to manufacturer’s protocol. Quantitative gene expression analysis was performed on an ABI PRISM 7700 (Applied Biosystems, Foster City, Calif) using SYBR Green technology. PCR thermal cycling conditions were 95°C for 30 sec, and 40 cycles of 95°C for 5 s and 60°C for 20 sec in a total volume of 20 µl/reaction. Transcript levels of target genes were calculated according to the 2^−ddCt^ method as supplied by the manufacturer (ABI PRISM 7700 user bulletin PE Applied Biosystems) and expressed in arbitrary units. The primers used are as follows: mouse NFATc1 primers: forward, 5′-GTGGCAGCCATCAACGCCCT-3′ and reverse, 5′- TACGAGGCCTGTGGCACCGA-3′; mouse β-actin primers: forward, 5′- TGGAATCCTGTGGCATCCATGAAAC-3′and reverse, 5′- TAAAACGCAGCTCAGTAACAGTCCG-3′; mouse IL-4 primers: 5′-TCGGCATTTTGAACGAGGTC-3′ and reverse, 5′-GAAAAGCCCGAAAGAGTCTC-3′; mouse INF-γ primers: 5′-CGCTACACACTGCATCTTGG-3′ and reverse:5′-TGAGCTCATTGAATGCTTGG-3′; mouse IL-2 primers:5′-GTGCTCCTTGTCAACAGCGC-3′and reverse: 3′-GAGCCTTATGTGTTGTAAGC-5′.

### Abs and Flow Cytometry

Control rat IgG (Cappel), and anti-CD3ε (2C11) were used for cell culture. Agonistic anti-mouse OX40 mAb (OX86) and inhibitory anti-OX40L mAb (RM134L ) were used to stimulate and inhibit OX40 signals, respectively.

For evaluation of NFATc1 expression, lymphocytes were first stained with PE- anti-CD4, then 1× permeabilizing solution (500 µL, Becton Dickinson) was added and incubated for 1.5 h at 4°C in the dark. After washing with 1 mL buffer, lymphocytes were stained with purified anti-NFATc1(7A6, santa cruz) followed by FITC–anti-mouse IgG (Sigma-Aldrich). Flow cytometry data were acquired on a FACSCalibur (BD Biosciences) and analyzed with Flow Jo software (Version 8.5.2; TreeStar).

### ELISA

Cultured supernatants were assessed for IL-2, IL-4 and IFN-γ cytokine content by enhanced sandwich ELISA protocols(eBioscience, Belgium).

### Statistical Analysis

Data were expressed as means ± SD and analyzed by SPSS 13.0 software. For comparisons between two variables, the unpaired Student's *t* test was used. Comparisons among more than two groups involved one-way analysis of variance (ANOVA) followed by Post Hoc LSD test. A two-tailed *P*<0.05 was considered statistically significant.

## Results

### Effects on the Atherosclerotic Plaques Area and Histological Aspects

**Figure 1:**
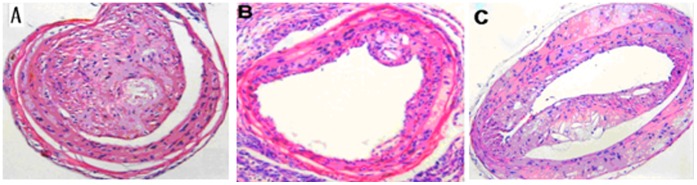
HE-stained cross sections of carotid 6 weeks in ApoE^−/−^ mice injecting with anti-OX40(100 µg/ml) and anti-OX40L(100 µg/ml) 2 times a week. Representative of plaques in control(C) and treated with anti-OX40(A) and anti-OX40L(B) stained with HE.

As shown in Fig. 1, HE staining revealed that the structures of carotid artery were different in ApoE^−/−^ mice. The model mice were injected with anti-OX40(100 µg/ml) and anti-OX40L(100 µg/ml) 2 times a week for 6 weeks, respectively. The plaques surface area was significantly increased in anti-OX40 treated group (Fig. 1A) compare with control group (Fig. 1C). Contrary, plaques in anti-OX40L treated group (Fig. 1B) were markedly decreased when compared with control and anti-OX40 treated group.

### OX40-OX40L Interaction and NFATc1 are Required for the Proliferation of Lymphocytes

**Figure 2:**
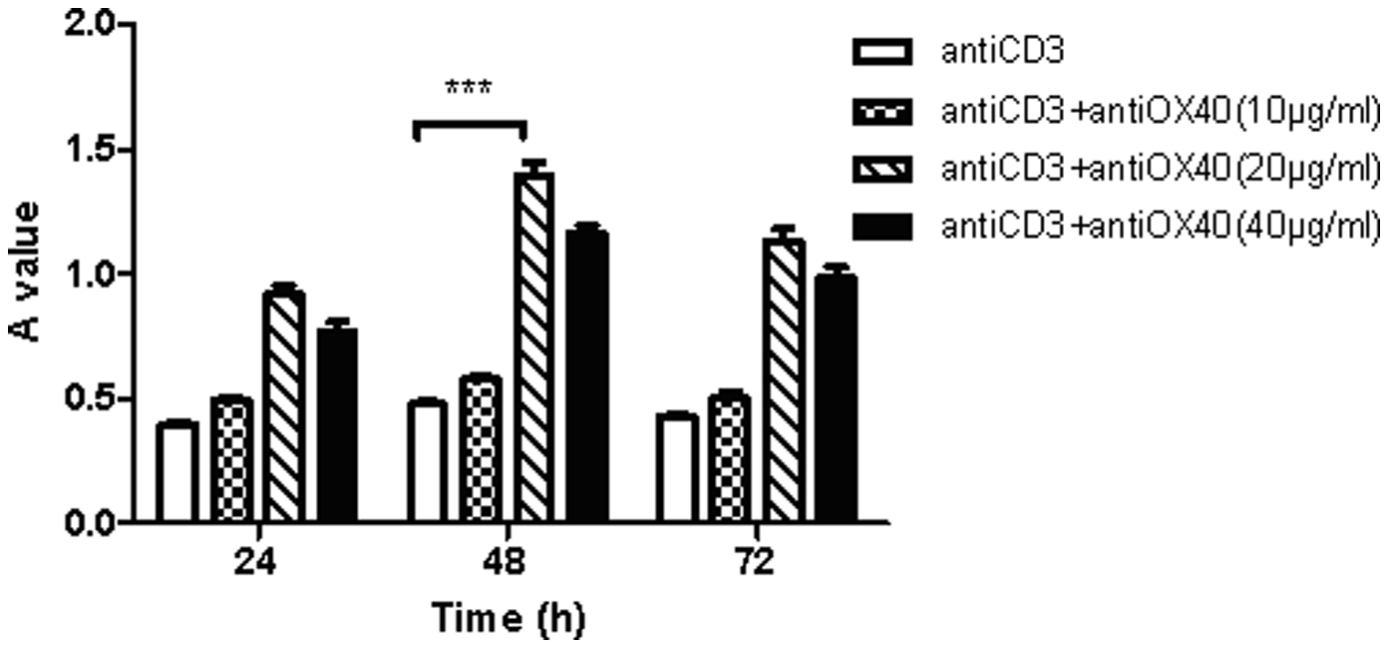
The proliferation of lymphocytes at 24 h, 48 h and 72 h after stimulated OX40-OX40L interaction by anti-OX40 (10, 20, 40 µg/ml). ***p<0.001 compared to respective control.

The effect of OX40-OX40L interaction on cell proliferation was measured using the MTT assay. First, lymphocytes were treated with a range of anti-OX40 (10, 20 and 40 µg/ml) and were harvested at 24 h, 48 h and 72 h. Anti-OX40 promoted proliferation of lymphocytes in time-dependent manner, reached maximum at 48 h, with maximal effect occurring at 20 µg/ml (Fig. 2), and the proliferation of lymphocytes didn’t show been promoted more effectively when were cultured with anti-OX40(40 µg/ml) or harvested at 72 h.

**Figure 3:**
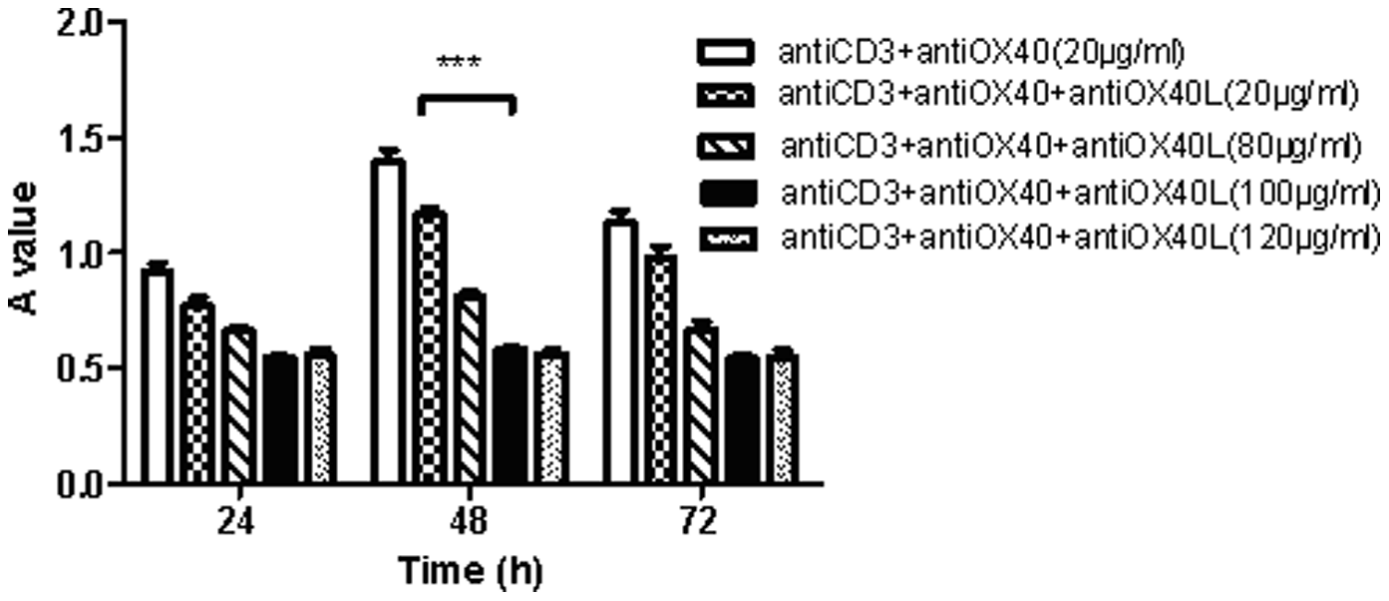
The proliferation of lymphocytes at 24 h, 48 h and 72 h after inhibition of OX40-OX40L interaction by anti-OX40L mAb (20, 80, 100 and 120 µg/ml). ***p<0.001 compared to control.

In pretreatment with anti-OX40L (20, 80, 100 and 120 µg/ml) for 10 min, the proliferation of lymphocytes induced by anti-OX40 is markedly suppressed, with maximal effect occurring at 100 µg/ml and 48 h (Fig. 3).

**Figure 4:**
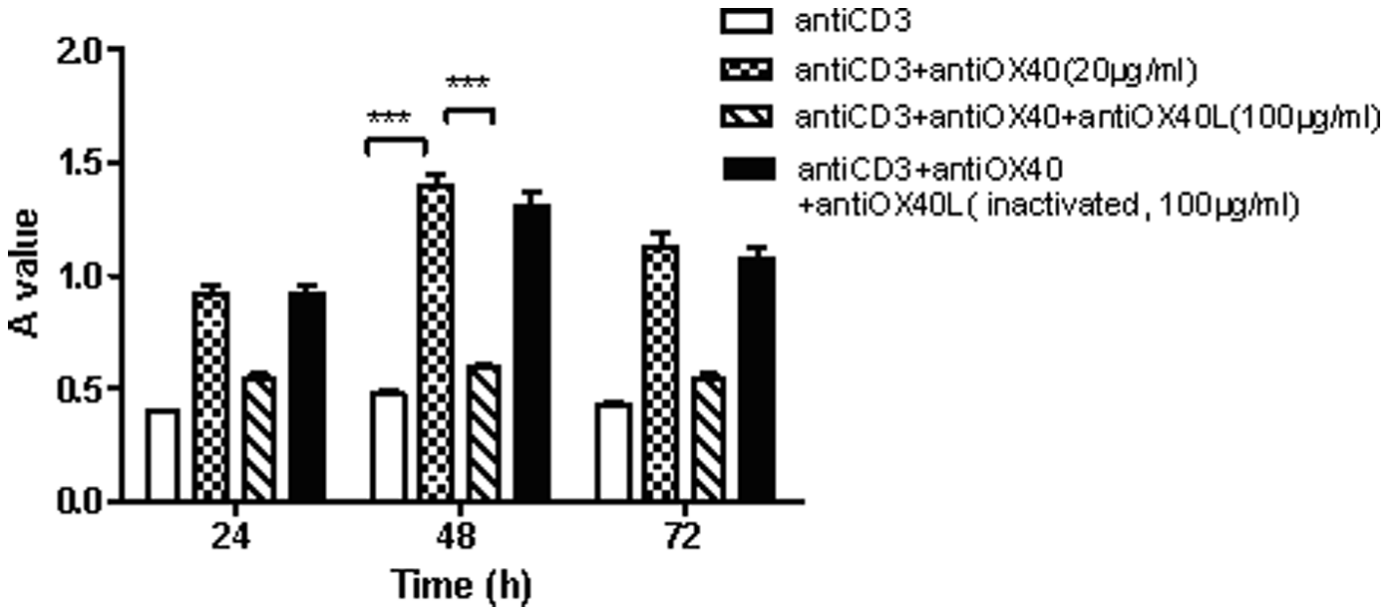
The proliferation of lymphocytes after interfered OX40-OX40L interaction. ***p<0.001 compared to control.

**Figure 5:**
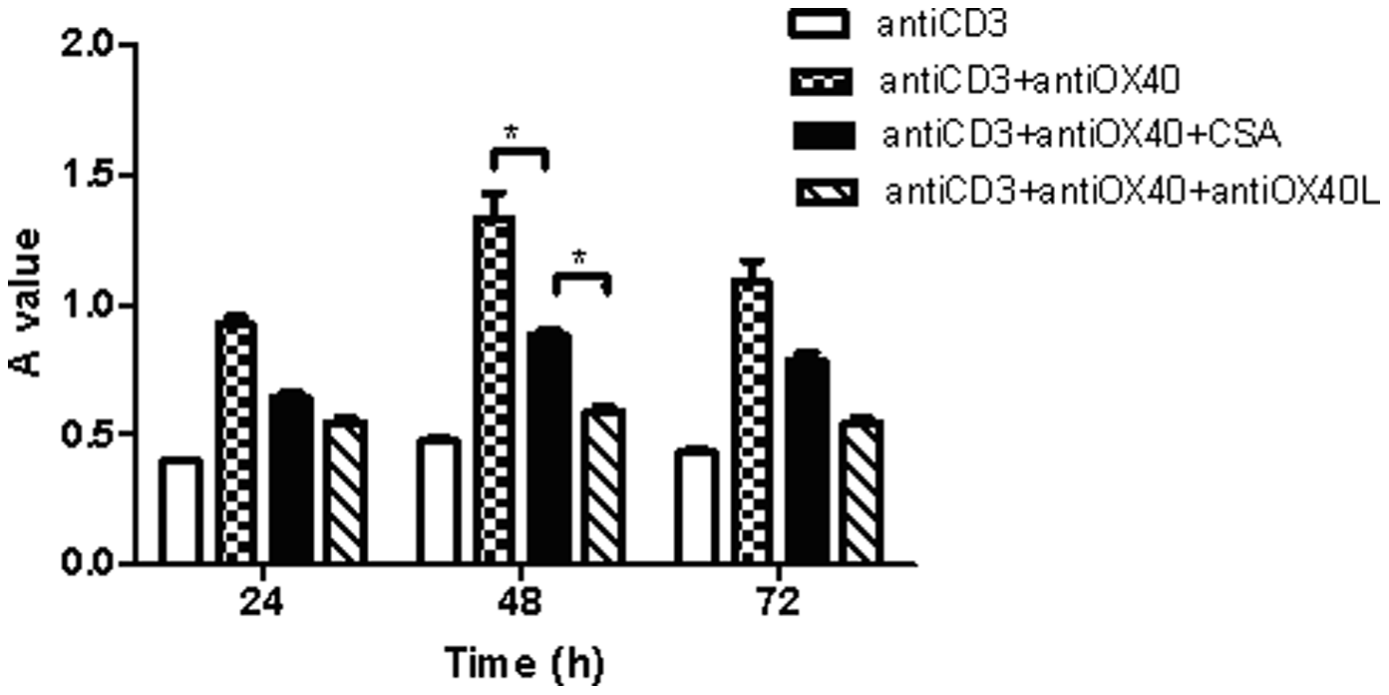
The proliferation of lymphocytes after interfered OX40-OX40L interaction. *p<0.05 compared to control.

Lymphocytes were treated with anti-OX40 (20 µg/ml), anti-OX40L (100 µg/ml) and inactivated anti-OX40L (100 µg/ml) respectively, at the maximal effect time (48 h), the proliferation of lymphocytes was markedly increased after anti-OX40-stimulated. Whereas down regulated after inhibited OX40-OX40L interaction, there was no difference between with or without inactivated anti-OX40L (Fig. 4). Anti-OX40L (20 µg/ml) or CsA(100 ng/ml) can significantly suppressed OX40-induced proliferation of lymphocytes (Fig. 5).

### OX40-OX40L Interaction on the Expression of NFATc1 in Lymphocytes

**Figure 6:**
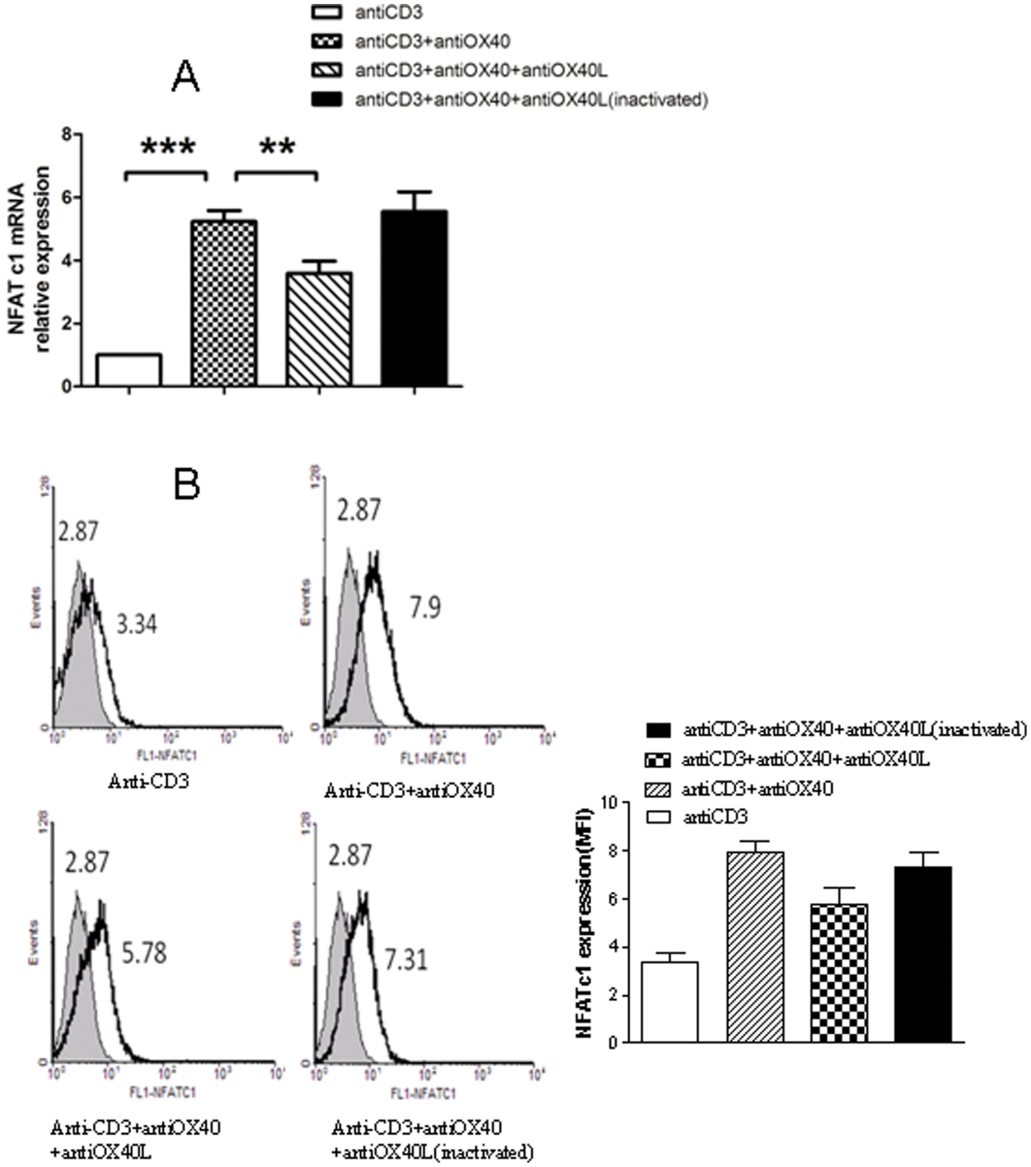
The expression of NFATc1 in lymphocytes by Real-Time PCR (A) and FCM (B). **, *** represent *p*<0.01 and *p*<0.001, respectively.

After cultured with anti-OX40 in the presence or absence of inactivated anti-OX40L, the expression of mRNA and protein of NFATc1 in lymphocytes is substantially up-regulation. In pretreatment with anti-OX40L(100 µg/ml) for 10 min, the expression of NFATc1 induced by anti-OX40 is markedly down-regulation (Fig. 6A,B).

### OX40-OX40L Interaction Controls Th Cell Activation through NFATc1 in Lymphocytes

**Figure 7:**
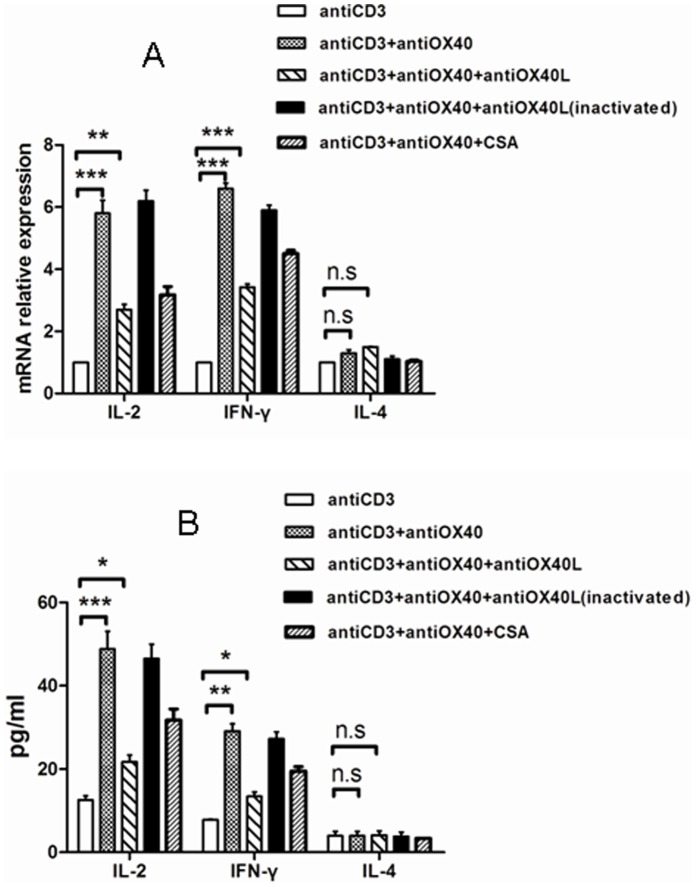
The levels of IL-2, IFN-γ and IL-4 induced by OX40-OX40L interaction in lymphocytes. *p<0.05, **p<0.01, ***p<0.001, ns *p*>0.05, respectively.

The expression of IL-2 and IFN-γ, in the cell and supernatants, was significantly increased when incubated with anti-OX40 in the presence or absence of inactivated anti-OX40L. A large decrease after anti-OX40-stimulated in the presence of anti-OX40L or CsA, but the level of IL-2 and IFN-γwas still more than that of treatment with anti-CD3 alone. However, for the same treatment, the expression of IL-4 in the cells and supernatants remained substantially unchanged (Fig. 7A,B).

### The Characteristics of Lymphocytes (Th)

**Figure 8:**
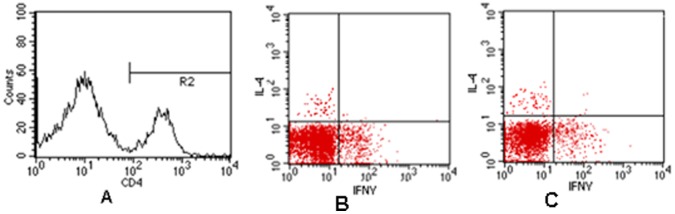
R2 were CD4^+^ lymphocytes (A). Th1 cells were labeled with IFNγ CD4^+^ lymphocytes. Th2 cells were labeled with IL-4 CD4^+^ lymphocytes. The proportion of Th1 and Th2 cells in the lymphocytes isolated from spleen of ApoE**^−/−^** mice(B). When lymphocytes were stimulated with anti-OX40, Th1 cells were significantly increased in lymphocytes(C).

We analyzed the lymphocytes with FACS during with or without anti-OX40-stimulated. As show in the Fig. 8, R2 were CD4^+^ lymphocytes (A). Th1 cells were labeled with IFNγ CD4^+^ lymphocytes. Th2 cells were labeled with IL-4 CD4^+^ lymphocytes. The proportion of Th1 cells were more than Th2 cells in the lymphocytes isolated from spleen of ApoE**^−^** ^/**−**^ mice (B). When lymphocytes were stimulated with anti-OX40, Th1 cells were significantly increased in lymphocytes (from 8.6% to 17.4%, C).

## Discussion

In our previous study, we found the OX40/OX40L interaction regulated the expression of NFATc1 in ApoE^−/−^ mice and promoted the intracellular signal transduction pathways [10]–[11]. OX40-OX40L is a pair of type II membrane glycoprotein with homology to the tumor necrosis factor family. OX40-OX40L interaction not only plays an important role in immune responses, but also may contribute to promotion of lymphocytes activation in the process of atherosclerosis.

Based on our previous researches, we investigated whether OX40-OX40L interaction regulated lymphocytes proliferation and viability through NFATc1 in ApoE^−/−^ mice. In this study, when stimulating OX40-OX40L interaction, the plaques surface area and proliferation of lymphocytes was significantly increased. Blocking OX40-OX40L interaction or using inhibitor of NTATc1(CsA), we found the similarly suppressed effect of the cells proliferation and plaques surface area. These data suggested that OX40-OX40L and NFATc1 may be act a critical role in the proliferation of lymphocytes and atherosclerotic plaques.

Most of the T lymphocytes present in the atherosclerotic plaques both in humans as well as in animal models were seen to be CD4^+^ T cells. These CD4 bearing Th cells have a central regulatory role in immune and autoimmune responses and are further classified mainly into Th1 subtype and Th2 subtype according to the cytokines they secrete. Studies in experimental models show a pro-atherogenic role for Th1 cells and an anti-atherogenic role for Th2 cells [13]–[14]. OX40-OX40L interaction has been linked to both Th1 and Th2 activation in several disease models [15]–[24]. We determined the capability of lymphocytes to produce Th1(IFN-γ,IL-2) and Th2 (IL-4) cytokines.

Our previous data shows that the OX40-OX40L interaction is transmitted through increasing the level of NFATc1 that accumulates and/or persists in the nucleus [11]. In this study, stimulating OX40-OX40L interaction, the expression of IL-2 and IFN-γwas significantly increased in cultured lymphocytes. While blocking the OX40-OX40L interaction or using inhibitor of NFATc1, we can see the similarly down-regulated effect on the expression of IL-2 and IFN-γ. However, the expression of IL-4 in supernatants remained substantially unchanged. Further analyzed with FACS, Th1 cells were significantly increased in lymphocytes when stimulated with anti-OX40.These data supports the opinion that atherosclerosis is a Th1-mediated disease. However, to clarify the precise mechanisms for these observations, further studies will be required.

From above results, OX40-OX40L and/or its downstream signaling intermediates such as NFATc1 will likely prove to be excellent future therapeutic targets for atherosclerosis. It is intruding to suggest that specific and early intervention directed at the interaction of OX40-OX40L and blockade of NFATc1 activation signaling might be a promising new approach to atherosclerotic disorders. They offer the advantage of a potentially selective immune-modulatory rather than harsh anti-inflammatory therapy, a strategy likely to be favourable in the context of atherosclerosis as a chronic inflammatory condition.
